# Swedish Nurses Pain Assessment Documentation of Children in the Perioperative Period

**DOI:** 10.1002/pne2.70041

**Published:** 2026-07-07

**Authors:** Emma Westin, Angelica Wiljén, Johanna Hagman, Stefan Nilsson

**Affiliations:** ^1^ Department of Health and Caring Sciences Linnaeus University Faculty of Health and Life Sciences Växjö Sweden; ^2^ Department of Paediatrics Region Kronoberg Växjö Sweden; ^3^ Centre of Interprofessional Collaboration Within Emergency Care (CICE) Växjö Sweden; ^4^ Institute of Health and Care Sciences, Sahlgrenska Academy University of Gothenburg Gothenburg Sweden; ^5^ Centre for Person‐Centred Care University of Gothenburg Gothenburg Sweden; ^6^ Södra Älvsborgs Hospital Borås, Region Västra Götaland Borås Sweden; ^7^ Department of Paediatrics Region Västra Götaland, Skaraborg Hospital Skövde Skövde Sweden; ^8^ Faculty of Caring Science, Work Life and Social Welfare University of Borås Borås Sweden; ^9^ Sahlgrenska University Hospital, Region Västra Götaland, Queen Silvia Children's Hospital Gothenburg Sweden

**Keywords:** children, pain assessment, pain prevalence, surgery

## Abstract

In healthcare, pain is common in children, with a higher risk of pain in children admitted for surgery. According to guidelines, pain should be assessed with a pain scale suitable to the age and maturity of the child to be able to provide the right treatment. However, research indicates shortages in assessment and documentation regarding children's pain. The aim of the current study was to describe the frequency and nature of pain assessment documentation, including pain intensity scoring, in surgical patients across the perioperative period. We performed a quantitative descriptive retrospective review of medical records at two county hospitals in the south of Sweden. In all, 114 children 7–17 years of age admitted to one of the hospitals in connection to surgery were included. Demographic and clinical data such as age, sex, type of surgery, and documentation regarding pain were collected from the medical records according to a predetermined protocol including the number and type of pain assessments and pain intensity scores. Data were analyzed with descriptive and inferential statistics. The findings show that pain scales were used and documented in 89 children (78.8%) once or more during the stay, and 155 (63.8%) of these assessments were performed in the recovery unit. One or more pain narratives were found in 113 (99.1%) of the medical records, of which 350 (69.6%) were written postoperative on the pediatric ward. There was a significant difference in the number of children who had at least one documented pain scale assessment, both between the hospitals and between the different time periods within the hospitals (preoperative on ward, recovery unit and postoperative on ward), but not between age groups. Moderate to severe pain was reported in 38.6% of the pain scale ratings. In conclusion, all the children had some kind of documented assessment of pain during their hospital stay, but not all children had a documented pain scale assessment. Documented pain scale assessments were most common in the recovery unit, whereas pain narratives were most common on the pediatric ward after the surgery. Looking at the entire length of the hospital stay, the result indicates that national guidelines for pain assessment are not being followed.

## 1 Introduction

In healthcare, pain is common in children [1, 2, 3, 4, 5], and the risk of pain is higher for children who are admitted for surgery [2, 4]. According to the SFAI (Swedish Society of Anaesthesiology and Intensive Care) guideline for acute and postoperative pain treatment in children, pain should be assessed with a pain scale suitable to the age and maturity of the child in order to be able to determine the right treatment. Postoperative pain should be assessed regularly, and when breakthrough pain occurs, the administered pain treatment should be evaluated [6]. However, pain scales are often underused in healthcare symptom assessments [1, 3, 4, 5, 7] even though a relatively high proportion of children within the healthcare system rate moderate to high pain when they are assessed [1, 2, 3, 5]. Previous research shows that it is inappropriate to replace the child's own pain assessment with that of healthcare personnel [5, 8] or parents [9, 10] since by proxy reports do not always correlate with the child's pain experience [5, 8, 9, 10].

Even though the child's own assessment of pain should constitute the foundation for decisions [8, 9, 10], many nurses seem to avoid using pain scales [11, 12]. Nurses often trust their clinical eye and do not think that the use of pain scales is necessary. It can also be perceived as complicated to assess pain in children since there are many different scales, and it can be difficult to trust the result if the child's assessment does not match their behavior [13]. However, to be involved in their care and be able to say what they want are the child's rights [14]. It can be hard to understand and express oneself in healthcare situations such as postoperative care. It is therefore important to regularly ask the child about symptoms in order to help them make their voice heard [15], especially since children do not always spontaneously tell healthcare personnel about their pain [1, 15].

In order to monitor pain and evaluate interventions, pain scale assessments need to be documented in the child's medical record. Nevertheless, documentation on pain is in many cases inadequate [1, 3, 4, 5, 7], and previous reviews of medical records have shown that it is common for healthcare personnel to use narrative pain documentation instead of pain scale ratings although sometimes it is used in combination [3, 4, 5]. To only use narrative pain documentation can be rich in content and clearly illustrate the child's pain, but in other cases it can be overly general with short notes and few details. It gives room for interpretation and uncertainty about how thoroughly the pain assessment was done [3].

Previous research thus indicates a shortage in assessment and documentation regarding children's pain [1, 3, 4, 5, 7]. Given that children admitted for surgery have an elevated risk of experiencing pain [2, 4], it is of utmost importance that guidelines on assessing and treating pain be followed. Therefore, this study was designed to describe the frequency and nature of pain assessment documentation, including pain intensity scoring, in surgical patients across the perioperative period.

## 2 Methods

### 2.1 Design and Settings

A descriptive retrospective review of medical records at two county hospitals (A and B) in the south of Sweden was conducted. Each hospital has a pediatric ward responsible for inpatient care for all the region's children independent of diagnosis. Both planned and emergency surgeries are performed. Local guidelines on pain assessment and treatment are available at hospital A, and they are consistent with the national SFAI guideline [6].

### 2.2 Sample

A consecutive sample of children and adolescents (henceforth “children”) aged 7 to 17 years (inclusive) who were admitted for surgery at one of the participating hospitals in connection to surgery received information about the study and were asked to take part. The information was either sent home with the appointment notice or issued on the pediatric ward after arrival; thereafter, healthcare personnel asked the child to participate.

### 2.3 Data Collection

Data collection, after permission from department managers, took place from November 2022 to July 2025. Demographic and clinical data such as age, sex, type of surgery, and documentation regarding pain were collected from the medical records by E.W. and J.H. according to a predetermined protocol including the number and type of pain assessments and pain intensity scores. The two types of pain assessment were either pain scale ratings using an evidence‐based pain scale where children self‐reported their pain or professionals rated their pain using an observational scale, or free text notations that is, pain narratives where the professional observed and asked the child about their pain. During the course of work, regular check‐ins were made with data comparisons to ensure that data collection was carried out equivalently at both hospitals. In total, 114 medical records were reviewed: 59 from region one and 55 from region two. Since 11 children were admitted to the pediatric ward after surgery, only 103 of 114 medical records involved both pre‐ and post‐operative time on the pediatric ward. One of these records was missing information about the time spent in the recovery unit. The remaining 11 medical records document the time in the recovery unit as well as the postoperative time on the pediatric ward; see Table 1.

**TABLE 1:** No. of entries from the three time periods.

Time period (*N* = 114)	Number of entries, *N* (%)
Preoperative on ward	103 (90.4%)
Recovery unit	113 (99.1%)
Postoperative on ward	114 (100%)

### 2.4 Data Analysis

Descriptive statistics are reported in absolute and relative frequencies (*n*, %). Complementary inferential statistical analyses were conducted. Comparisons in the number of children with at least one documented pain scale assessment between the hospitals and age groups were made with Fisher's exact test. Comparisons between the different time periods within the hospitals on the ward/in the recovery unit were made with Friedman test and Wilcox signed rank test. The statistical significance level was set at *p* < 0.05. The collected variables were analyzed with the SPSS version 30.0 (IBM Corp., Chicago, IL, USA).

### 2.5 Ethical Considerations

Children and the parents of children under 15 years of age obtained written and verbal information with the opportunity to ask questions. The written information letter provided information about voluntary participation and advised that collected data are treated confidentially in accordance with the Declaration of Helsinki [16]. Four versions of the information letter, adjusted to different age groups and one for the parents, were used. Children aged 14 years or younger gave verbal consent, and written consent was collected from their parents. Children aged 15 years up gave verbal and written consent. Ethical approval was obtained from the Swedish Ethical Review Authority (dnr: 2022‐03188‐01).

## 3 Results

### 3.1 Demographics

Approximately the same number of girls and boys participated (median age 12 years). Fifteen children from the younger age group and five from the older age group that initially consented to participation, were excluded due to the lack of a signed consent form. The majority of the surgeries were performed urgently (70.2%), with orthopedic surgery being most common (44.7%). Overall, 103 of the 114 children were admitted to the pediatric ward before their surgery, and their stay in hospital was divided into three periods: preoperative time on the pediatric ward, time in the recovery unit, and postoperative time on the pediatric ward. The majority of these children stayed < 12 h on the pediatric ward before surgery. The remaining 11 children stayed in the recovery unit and postoperatively on the pediatric ward. The most common length of stay in the recovery unit was 1–2 h, and most of the children were admitted for up to 1 day on the pediatric ward after surgery. For demographic data, type of surgery, urgency of the operation, and length of stay, see Table 2.

**TABLE 2:** Demographic data, type of surgery, urgency of operation, and length of stay.

*n* = 114	*N*	%
Age		
7–11 years	55	48.2
12–17 years	59	51.8
Sex		
Girls	53	46.5
Boys	61	53.5
Type of surgery		
Orthopedic (ORT)	51	44.7
General surgery (GS)	38	33.3
Ear, nose and throat (ENT)	24	21.1
Orthopedic + GS (ORT + GS)	1	0.9
Urgency		
Planned	34	29.8
Emergency	80	70.2
Admitted before surgery		
Yes	103	90.4
No	11	9.6
Length of stay		
Preoperative on ward (*n* = 103)		
< 12 h	64	62.1
12–24 h	31	30.1
> 24 h	8	7.8
Recovery unit (*n* = 113)		
< 1 h	8	7.1
1–2 h	74	65.5
> 2 h	31	27.4
Postoperative on ward		
< 12 h	16	14.0
12–24 h	63	55.3
> 24 h	35	30.7

### 3.2 Documented Pain Assessments

**FIGURE 1:**
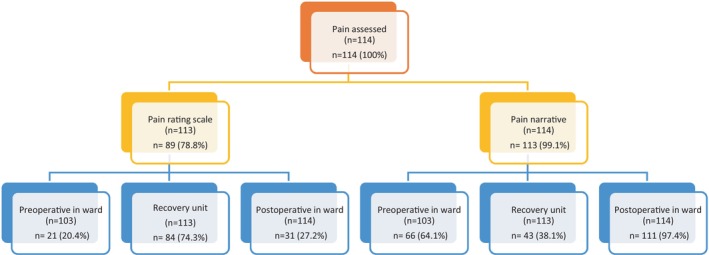
Assessments with a pain rating scale/pain narrative.

All children had at least one pain narrative that assessed their pain and/or a documented pain assessment with an evidence‐based pain scale. 97.1% of the assessments were self‐reported pain using VAS or NRS, and 2.9% of the assessments were done by the professionals with an observational pain scale called the Face, Legs, Activity, Cry, and Consolability (FLACC) scale (exclusively in the recovery unit). Pain scales were used with 89 children (78.8%) once or more during their stay, of whom the majority were assessed in the recovery unit. One or more pain narratives were found in 113 (99.1%) medical records (see Figure 1), and the majority were written during the postoperative time on the pediatric ward. There was a significant difference in the number of children with at least one documented pain scale assessment, both between the hospitals and between the different time periods within the hospitals (preoperative on ward, recovery unit, and postoperatively on ward), but not between age groups, see Table 3.

**TABLE 3:** Number of children with at least one pain assessment made with an evidence‐based pain scale.

	No. of patients (preop/recovery/postop)	No. of children with at least one pain scale assessment		
		Preop on ward	Recovery unit	Postop on ward
Hospital				
Region A^a^	(50/58/58)	19 (38.0%)	55 (94.8%)	22 (37.9%)
Region B^a^	(53/55/56)	2 (3.8%)	29 (52.7%)	9 (16.1%)
Total	(103/113/114)	21 (20.4%)	84 (74.3%)	31 (27.2%)
*p*		< 0.001	< 0.001	0.011
Age				
7–11 years	(52/55/55)	8 (15.4%)	37 (67.3%)	13 (23.6%)
12–17 years	(51/58/59)	13 (25.5%)	47 (81.0%)	18 (30.5%)
Total	(103/113/114)	21 (20.4%)	84 (74.3)	31 (27.2%)
*p*		0.230	0.131	0.528

^a^ Friedman test shows a significant difference (< 0.001) in the number of documented pain scale assessments made preoperatively on ward, in recovery unit, and postoperatively on ward, within each hospital. Wilcox Signed Ranks test shows a significant difference (< 0.001) in the number of documented pain scale assessments made in the recovery unit and postoperatively on ward within each hospital.

The number of documented pain assessments with a pain scale during the whole stay varied from none to 15 per child (Md 2). In total, pain scale assessments were used 243 times, of which 155 (63.8%) were in the recovery unit. The number of pain narratives varied between none and 29 per child (Md 3). In total, 503 pain narratives were found in the records; 350 (69.6%) of the notes were written postoperatively on the pediatric ward (see Table 4). A pain narrative could be based on how the child described their pain (e.g., “complains of dull pain” or “denies pain”). In other cases, it contained a more subjective observation, such as “is perceived to be very affected by pain” or “has not complained of pain.”

**TABLE 4:** Assessments with pain scale/pain narrative per time period and age group, type of surgery, and urgency.

	No. of patients (preop/recovery/postop)	No. of assessments with pain scale (Md, range)			
		Preop on ward	Recovery unit	Postop on ward	Total
Total	(103/113/114)	32 (0, 0–3)	155 (1, 0–12)	56 (0, 0–7)	243
Age					
7–11 years	(52/55/55)	14 (0, 0–3)	60 (1, 0–5)	27 (0, 0–7)	101
12–17 years	(51/58/59)	18 (0, 0–2)	95 (1, 0–12)	29 (0, 0–3)	142
Operation					
ENT	(20/24/24)	0 (0, 0–0)	32 (1, 0–4)	4 (0, 0–1)	36
Ort	(46/50/51)	11 (0, 0–2)	56 (1, 0–3)	24 (0, 0–7)	91
GS	(36/38/38)	20 (0, 0–3)	55 (1, 0–5)	26 (0, 0–3)	101
ORT + GS	(1/1/1)	1 (1, 1–1)	12 (12–12)	2 (2, 2–2)	15
Urgency					
Planned	(26/33/34)	0 (0, 0–0)	47 (1, 0–4)	10 (0, 0–2)	57
Emergency	(77/80/80)	32 (0, 0–3)	108 (1, 0–12)	46 (0, 0–7)	186

	No. of patients (preop/recovery/postop)	No. of pain narratives (Md, range)			
		Preop on ward	Recovery unit	Postop on ward	Total
Total	(103/113/114)	109 (1, 0–6)	44 (0, 0–2)	350 (2, 0–23)	503
Age					
7–11 years	(52/55/55)	48 (1, 0–3)	27 (0, 0–2)	139 (2, 0–11)	214
12–17 years	(51/58/59)	61 (1, 0–6)	17 (0, 0–1)	211 (2, 1–23)	289
Operation					
ENT	(20/24/24)	5 (0, 0–2)	12 (0.5, 0–1)	48 (2, 1–4)	65
Ort	(46/50/51)	54 (1, 0–3)	16 (0, 0–1)	157 (2, 0–18)	227
GS	(36/38/38)	44 (1, 0–5)	15 (0, 0–2)	123 (2, 0–23)	182
ORT + GS	(1/1/1)	6 (6, 0–6)	1 (1, 1–1)	22 (22, 22–22)	29
Urgency					
Planned	(26/33/34)	3 (0, 0–1)	14 (0, 0–1)	68 (2, 1–4)	85
Emergency	(77/80/80)	106 (1, 0–6)	30 (0, 0–2)	282 (2, 0–23)	418

### 3.3 Pain Ratings and Pain Intensity

**FIGURE 2:**
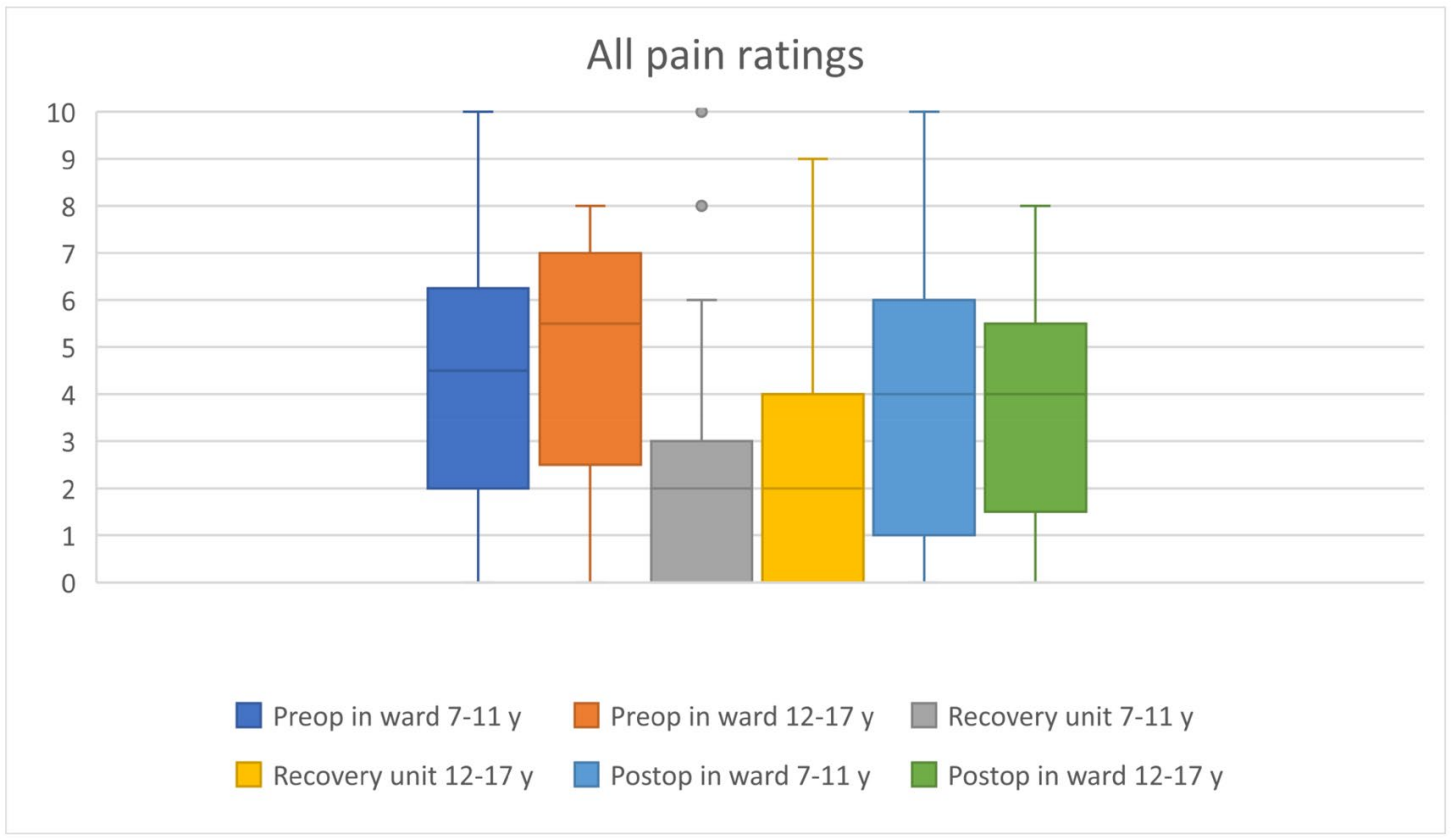
Pain ratings per age group and time period.

**FIGURE 3:**
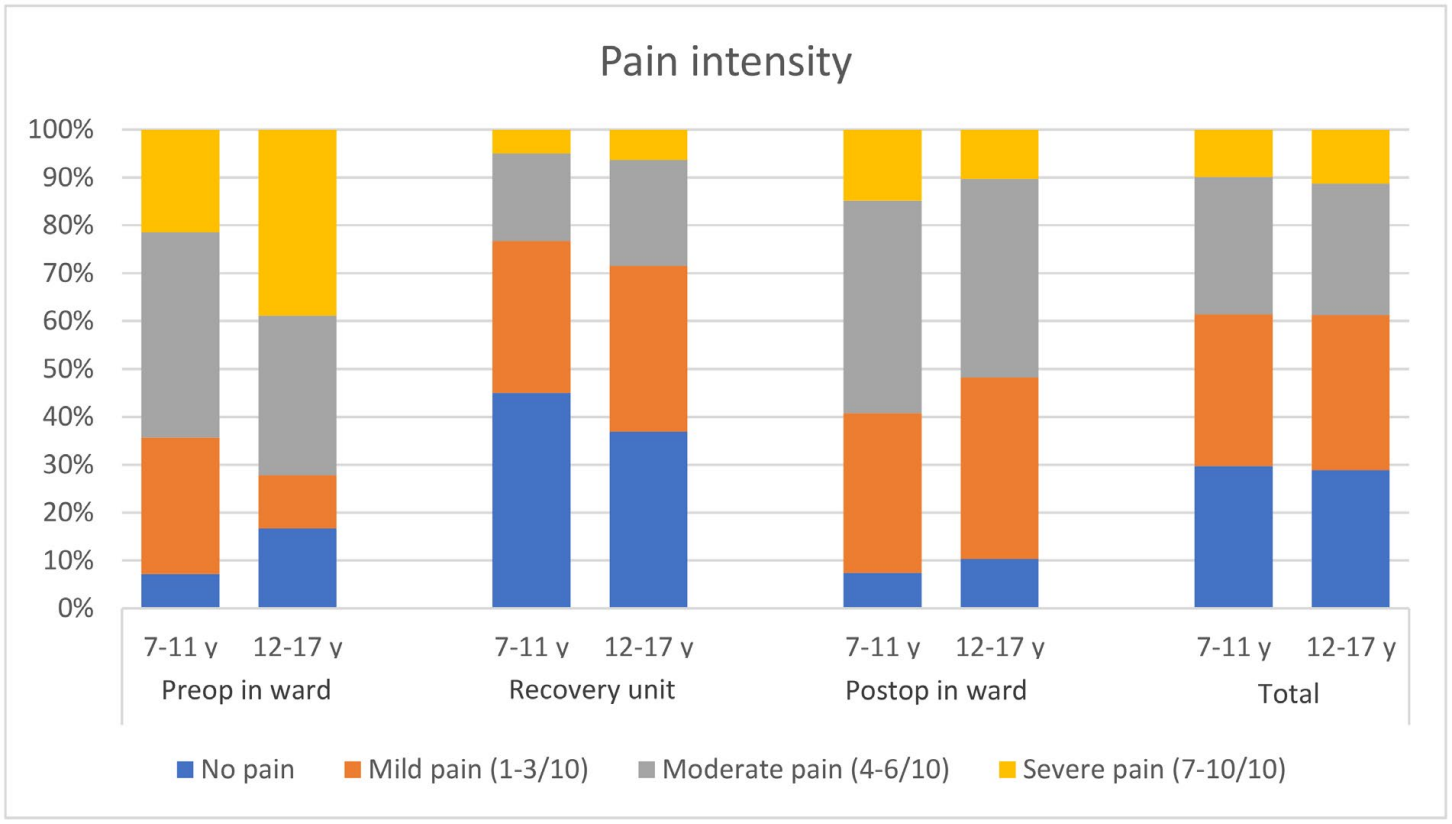
Pain intensity per age group and time period.

The evidence‐based pain rating scales used were either the Visual Analogue Scale (VAS), the Numeric Rating Scale (NRS), and in a few cases, the FLACC scale. These scales have ranges of 0–10. Overall, the median value for pain was 3 (range 0–10), which indicates mild pain, but variation can be seen in how the children's pain was rated. In 29.7% of the ratings, no pain was reported (0/10); 31.7% of the ratings reported mild pain (1–3/10), 28.7% moderate pain (4–6/10), and 9.9% severe pain (7–10/10). The pain ratings are displayed in age groups and time periods in Figure 2, with pain intensity illustrated in Figure 3.

## 4 Discussion

This study aimed to describe the frequency and nature of pain assessment documentation, including pain intensity scoring, in surgical patients across the perioperative period. The main results showed that all children had at least one pain narrative and/or a documented pain assessment during their perioperative period. The most common way to assess pain preoperatively and postoperatively on the ward was through pain narratives, while pain rating scales were most frequently used in the recovery unit. There was a significant difference in the number of children with at least one documented pain scale assessment, both between the hospitals and between the different time periods within the hospitals (preoperative on ward, recovery unit and postoperative on ward), but not between age groups. On average, the children rated their pain as 3 on a 10‐point scale; however, nearly 38% of the children reported moderate to severe pain at some point during their hospital stay.

Even though pain is subjective and can vary between individuals and types of procedure, children admitted for surgery have a particularly high risk of experiencing significant pain [2, 4, 17, 18, 19, 20]. With that in mind, as well as the fact that the national guideline advocates that regular pain scale assessments be made in relation to surgery, this is a context where one should find a high prevalence of pain assessments compared to non‐surgical care. Previous research has shown variation in terms of how frequently pain scale assessments are documented, with numbers ranging from slightly under 40% to over 95% [1, 2, 3]. It has also shown that children's pain is not evaluated enough; specifically, young children and children with impairments are under‐assessed [21, 22, 23, 24]. Looking at the whole length of stay, the majority of the children (78.8%) in this study had at least one pain scale assessment documented in their medical record. This could be considered an acceptable result. At the same time, it means that 21.2% of the children did not have the opportunity to have their pain rated (neither by themselves nor by the personnel).

According to the findings there were no significant differences between the number of children with at least one documented pain scale assessment between the two age groups, as has been shown in previous studies where older children tend to be more assessed. One reason for younger children to be less assessed might be due to communication and developmental issues [25]. One other reason might be that it is more time‐consuming to help young children understand the scale used for assessing pain and also due to the inexperience of the way children in different ages address pain and the professionals often assuming that the children will tell if they are in pain [15]. However, in this study the youngest children were 7 years old which could offer a possible explanation for why the difference was not observed since they were a bit older than the younger children often referred to in previous research. It should also be noted that 63.8% of the total 243 pain scale assessments were performed in the recovery unit and, in other words, only about 20%–30% of the children on the ward had documentation of at least one pain scale assessment in their medical record before or after their surgery. There was a significant difference in the number of children with at least one documented pain scale assessment on the pediatric ward and in the recovery unit in both hospitals. That some of the surgeries were planned could to some extent explain why there were fewer documented pain scale ratings before surgery; however, approximately 70% of the surgeries were urgent.

It has been observed that parents and children can be satisfied with pain treatment even though the child has not been subjected to pain scale assessment to any great extent [4, 5]. Treatment satisfaction appears to be associated with other values, such as feeling listened to and having one's concerns acknowledged [5]. Also, the absence of an assessment with pain scales does not mean that the child did not receive any kind of pain assessment during their stay. When Skog et al. [13] interviewed nurses on their view on pain assessment, it appeared that they considered it a natural part of postoperative care, and asking children and parents about pain felt relevant and appropriate, but the need to use a pain scale was not obvious [13]. Almost all the children in this study (99.1%) had at least one pain narrative in their medical record, of which the majority (69.6%) were written postoperatively on the pediatric ward. That is a higher proportion than has been reported in previous research; however, those reports included all admitted children and not only those who had surgery [1, 3, 4, 5]. The finding indicates that nurses were aware of and talked to the children about pain. The fact that there are more pain narratives than ratings with a pain scale may be due to nurses perceiving scales as complicated to use with children [12, 13]. It can feel easier to just ask the child without using a pain scale and, based on their response, form an impression on the pain [12]. The choice does not appear to be due to a lack of knowledge; previous research shows that nurses' knowledge about pain rating is sufficient [11, 12]. This is confirmed by the fact that education interventions around pain assessment do not always seem to have much effect on the use of pain scales [26]. Another explanation is that it could be seen as adding to the workload to perform pain scale assessments, and some nurses have reported that clear guidelines around pain scales are missing [13]. However, when this study was performed, a national guideline and, at one of the hospitals, a local guideline was in place. If all nurses had knowledge of the guideline(s) is unknown, as are the reasons one was not followed. Nevertheless, this study's result showed a significant difference between the number of children with at least one documented pain scale assessment between the hospitals, with a higher proportion at the hospital having a local guideline about pain assessment and treatment. At the same time there also were significant differences between the pediatric ward and the recovery unit within both hospitals. This indicates that assessing and documenting pain can be considered a complex task, not only regulated by guidelines. Yuan [26] suggests that there can be both organizational, educational and cultural factors to consider [26]. Regardless, this study does not give an answer to how much impact the local guideline had on the results. Another reason for not using pain scales is that it can be tempting to think that a pain narrative is sufficient but, as Stevens et al. [3] pointed out, it gives space for interpretation, which can complicate assessment, treatment, and evaluation of the child's pain. It should, however, be noted that even though pain treatment outcome often is measured in the number of pain assessments and documentation about pain, these things also need to lead to action to relieve the pain. In other words, there seems to be no guarantee that pain assessments lead to good pain control [27]. Furthermore, the fact that children and caregivers are typically satisfied with the pain treatment regardless of pain scale assessments [4, 5] sparks curiosity with respect to their expectations and wishes regarding pain management because, for them, there is obviously more to it than what guidelines state.

There are nonetheless good reasons for making pain scale assessments. When Senger et al. [28] let admitted children rate their highest pain value in the last 24 h, it turned out that the children reported higher values than were documented in their medical records from earlier pain scale assessments [28]. Also, researchers often find a high proportion of children who report moderate to severe pain when they are in hospital. This indicates that there is a risk of missing a child in need of treatment if pain scale assessments are not made regularly. However, the variation between studies is high. The prevalence of children with moderate to severe pain extends from slightly below 25% to nearer 80% depending on the study's context and which children were included [1, 2, 3, 5, 27]. Children who are assessed with a pain scale in connection to surgery rate, not surprisingly, higher values [2, 4]. That said, it must be noted that pain is subjective, which makes it hard to compare pain intensity since the values have different meanings for different children [29]. In this study, the proportion of ratings ≥ 4 on a 10‐point scale was nearly 38%, but it is difficult to draw any conclusions on how many of those children needed pain treatment, as the cut‐off value for the indication can differ from child to child. Some children rate with high values but do not feel they need pain treatment, and vice versa [29]. This could contribute to some nurses finding it hard to rely solely on pain scale assessments [13]. An overly rigid way of thinking around which values count as mild, moderate, or severe pain has a risk of creating mistrust against the pain scale itself. That is an argument for why including the child in their own care is so important when it comes to assessing and treating pain. By talking to the child, their individual limits for what is considered “acceptable” pain can be revealed. These limits should be considered when making treatment decisions.

## 5 Strengths and Limitations

A strength of this study is that a joint protocol was used to ensure that the reviews were performed in a similar manner. Another strength is that it includes the whole length of stay and not just a sample of it. A limitation is that not all children were admitted before their surgery, which makes it harder to make comparisons between the three groups. Another aspect of that is that we included children undergoing different types of surgery. At the same time, this is the reality of pre‐ and post‐operative care in smaller hospitals, where all children are admitted to the same ward. A further strength is that the study was conducted at county hospitals when many previous pediatric studies were conducted in larger settings such as university hospitals. No children under the age of 7 years were included, which could have affected the results since they are less likely to be assessed with a pain rating scale than older children [11].

## 6 Conclusion and Implications

This study was designed to describe the frequency and nature of pain assessment documentation, including pain intensity scoring, in surgical patients across the perioperative period. The findings show that all children had some kind of documented assessment of their pain during their stay in hospital, but not all children had a documented pain scale assessment. Documented pain scale assessments were most common in the recovery unit, whereas pain narratives were most common on the pediatric ward. Looking at the entire length of the hospital stay, the results indicate that the SFAI guideline for pain assessment is not being followed.

Following the guideline and using pain scales for pain assessment are necessary to avoid missing a child experiencing pain. But it is not clear that the use of pain scales leads to parents and children feeling more satisfied with the pain treatment. Pain ratings cannot stand on their own; they need to be completed with the child's own view on what the different values mean. Combining the use of pain scales with pain narratives allows the possibility to create an overall picture that is greater than the sum of its parts. Future research could focus on understanding what is needed to ensure that the existing guideline is followed, including how parents and children can be made more involved and participate more actively in pain assessments.

## Conflicts of Interest

The authors declare no conflicts of interest.

## Data Availability

The data that support the findings of this study are available from the corresponding author upon reasonable request.
